# Night eating syndrome (NES). A case report of NES

**DOI:** 10.1192/j.eurpsy.2023.1796

**Published:** 2023-07-19

**Authors:** B. Pascual Garcia, C. Boix Abad, D. Vazquez Gonzalez

**Affiliations:** 1 Mental Health Center, CPB, Barcelona, Spain

## Abstract

**Introduction:**

The night eating syndrome (NES, DSM-V: 307.59) was described in 1955 as a disorder defined by morning anorexia, nocturnal hyperphagia (25% of the daily intake of food during sleep) and insomnia. Attributed to a delay in the circadian rhythm of feeding is characterized by suppression of the daytime appetite and increased in the early morning. It is more prevalent in obese people. Treatment focuses on selective serotonin reuptake inhibitors (SSRIs) and cognitive behavioural therapy (CBT).

**Objectives:**

Description of a NES clinical case demonstrated satisfactory response to pharmacological treatment with trazodone.

**Methods:**

Brief case presentation and review of the NES literature.

**Results:**

A 40-year-old woman diagnosed with binge eating disorder followed by Endocrinology. She had morbid obesity grade III. After the failure of various treatments addressed to impulse control and early morning intakes (topiramate, zonisamide, liraglutide, gastric balloon, hydrochloride Naltrexone/Bupropion and SSRI) she was referred to a mental health center. She was started on Trazodone therapy. Interestingly, Insomnia/binge decreased from 7 to 2 episodes/ week leading to a significant weight reduction with a 500 mg/week rate, loosing 4 kg. The case was oriented as a NES but successfully treated as maintenance insomnia.

**Conclusions:**

NES leads to frequent awakenings and early morning intakes. Awareness of the episode and ability to remember differentiates NES from the sleep-related eating disorder, where the nighttime ingestions cannot be remembered. Unlike binge eating disorder, where the goal is satiety and not falling asleep, so the hypnotic function of nocturnal intake is key in the differential diagnosis with NES . Trazodone, indicated in insomnia of maintenance, has not been reported as a treatment of choice in the NES, enabling a significant decrease on awakenings and consequently the intake.

**Disclosure of Interest:**

None Declared

